# Needs and barriers to improve the collaboration in oral anticoagulant therapy: a qualitative study

**DOI:** 10.1186/1471-2261-11-76

**Published:** 2011-12-22

**Authors:** Hanneke W Drewes, Mattijs S Lambooij, Caroline A Baan, Bert R Meijboom, Wilco C Graafmans, Gert P Westert

**Affiliations:** 1 Scientific Centre for Care and Welfare (Tranzo), Tilburg University, Tilburg, The Netherlands; 2 Centre for Prevention and Health Services Research, National Institute for Public Health and the Environment, Bilthoven, The Netherlands; 3 Scientific Institute for Quality of Healthcare (IQ healthcare), Radboud University Nijmegen Medical Centre, Nijmegen, The Netherlands

## Abstract

**Background:**

Oral anticoagulant therapy (OAT) involves many health care disciplines. Even though collaboration between care professionals is assumed to improve the quality of OAT, very little research has been done into the practice of OAT management to arrange and manage the collaboration. This study aims to identify the problems in collaboration experienced by the care professionals involved, the solutions they proposed to improve collaboration, and the barriers they encountered to the implementation of these solutions.

**Methods:**

In the Netherlands, intensive follow-up of OAT is provided by specialized anticoagulant clinics (ACs). Sixty-eight semi-structured face-to-face interviews were conducted with 103 professionals working at an AC. These semi-structured interviews were transcribed verbatim and analysed inductively. Wagner's chronic care model (CCM) and Cabana's framework for improvement were used to categorize the results.

**Results:**

AC professionals experienced three main bottlenecks in collaboration: lack of knowledge (mostly of other professionals), lack of consensus on OAT, and limited information exchange between professionals. They mentioned several solutions to improve collaboration, especially solutions of CCM's decision support component (i.e. education, regular meetings, and agreements and protocols). Education is considered a prerequisite for the successful implementation of other proposed solutions such as developing a multidisciplinary protocol and changing the allocation of tasks. The potential of the health care organization to improve collaboration seemed to be underestimated by professionals. They experienced several barriers to the successful implementation of the proposed solutions. Most important barriers were the lack motivation of non-AC professionals and lack of time to establish collaboration.

**Conclusions:**

This study revealed that the collaboration in OAT is limited by a lack of knowledge, a lack of consensus, and a limited information exchange. Education was identified as the best way to improve collaboration and considered a prerequisite for a successful implementation of other proposed solutions. Hence, the implementation sequence is of importance in order to improve the collaboration successfully. First step is to establish alignment regarding collaboration with all involved professionals to encounter the lack of motivation of non-AC professionals and lack of time.

## Background

Oral anticoagulant therapy (OAT) - provided to prevent thrombosis - is one of the major causes of drug related avoidable hospitalizations [1,2]. The use of oral anticoagulants is risky in itself because anticoagulants have a narrow therapeutic range requiring a careful balancing between the risk of haemorrhage and the risk of thrombosis [3]. This delicate balance is easily affected by factors such as co-prescription or dosage modification [4,5]. Consequently, many health care professionals are involved in OAT all of whom can potentially influence the effect of the therapy [6]. Therefore, collaboration between professionals is expected to be essential in order to prevent adverse events from happening.

Since oral anticoagulants are frequently used over a long period of time, OAT requires a chronic care approach. With respect to OAT, differences in chronic care management exist between countries. Chronic care management is provided by routine medical care (for instance in France and the US) or by specialized anticoagulant clinics (ACs) (for instance in Italy and the Netherlands). The characteristics of OAT provided by the ACs in the Netherlands are outlined in table 1.

**Table 1:** Characteristics of OAT provided by Dutch ACs [8,12,28]

Characteristic	Dutch context
History	The first AC was introduced in 1949.
Number	61 ACs manage OAT for all outpatients in the Netherlands.
Organization	Substantial organizational variations exist between ACs such as the location (within or outside the hospital), the legal form (independent organization or department of hospital or GP-lab), and the number of patients (about 1100 to 21500 patients).
Chronic care management	The International Normalized Ratio (INR) - a standardized transformation of the prothrombin time - is used to determine the dosage of oral anticoagulants needed to correct the prothrombin time.
	The management of OAT by specialized nurse and/or physicians of all ACs implies: measuring INRs, gathering relevant patient information, providing patient education and self-management support, and giving dosage advices.
	Patients on oral anticoagulants consult an AC at least once every 6 weeks.
Additional tasks	ACs can perform additional tasks such as giving dosage advices before surgery, giving dosage advices for patients living in nursing homes, and giving dosage advices to hospitalized patients.
Collaboration	Collaboration varies between ACs. For instance, about 55% percent of ACs have formal agreements with at least one hospital, 19% use a webbased clinical information system, and less than half of all ACs are always informed about interacting drugs by the pharmacist.

Even though there is a risk that ACs, being yet another actor in OAT, could lead to more fragmented care, studies show that because of the pivotal role they play in OAT, they achieve better patient outcomes than routine medical care [3,7]. However, substantial variation in organization and patient outcomes between Dutch ACs is prevalent. For instance, 55% percent of ACs have formal agreements with at least one hospital and 19% use a webbased clinical information system [8]. Since organizational characteristics are assumed to influence the collaboration [9-11], it can be expected that the extent of collaboration between AC regions will differ causing differences in quality of care between regions. In order to reduce this variation and improve the collaboration between care services across the care continuum, insight is needed into the needs and mechanism to improve the collaboration in OAT. This study aims first to identify the bottlenecks in the collaboration between health care professionals in OAT as experienced by managers, medical specialists, and nurses. Second, it aims to identify the solutions proposed to improve collaboration and the barriers to the implementation of these proposed solutions, with the ultimate aim to find out what is required to improve the quality of OAT.

## Methods

### Setting and study participants

Because intensive OAT follow-up is provided by ACs in the Netherlands [12], we focused our interviews on the experiences of the AC professionals. We performed an a-selective purposive sampling procedure based on three characteristics of the 61 Dutch ACs: number of patients, organisation (ACs as a hospital department or, affiliated with a GP-laboratory, or independent ACs), and quality of care (operationalised as the percentage of patients within the correct therapeutic range). Within each category we randomly selected 30% of the ACs, resulting in 23 ACs.

At each AC, a trained interviewer (HD) conducted interviews with at least one director or manager, one medical doctor and/or quality manager, and one nurse (dosage advisor and/or employee who takes blood samples and collects patient information). We conducted a total of 68 face-to-face interviews with 103 professionals (16 directors, 28 medical doctors, 17 quality managers, 26 dosage advisors, and 14 employees who take blood samples and collect patient information). The semi-structured interviews took place between November 2008 and February 2009. All interviews were audio-recorded with permission of the participants and subsequently transcribed verbatim.

The following themes - covered by the semi-structured interview protocol (see additional file 1) - were identified: 1) collaboration experiences with other professionals; 2) proposed solutions to improve collaboration; 3) perceived barriers to the successful implementation of the proposed solutions.

### Data analysis

The three themes - bottlenecks, proposed solutions, and perceived barriers - were independently studied and open coded by the first two authors (HD and ML) using a template. This template was inductively created during the coding process and used by both researchers to index all 68 transcripts independently. Inconsistencies were resolved by consensus. Differences between the types of professionals (i.e. manager, doctor, and nurse) were analyzed.

All the proposed solutions were categorized by the authors according to Wagner's chronic care model (CCM) (table 2). The CCM is used worldwide to improve chronic care which includes the improvement of collaboration between professionals as one of its fundamental goals. Wagner's model recognizes the multidimensionality of collaboration: collaboration is influenced by many factors such as shared goals and vision, leadership and information exchange [9-11,13]. In particular, the CCM includes four components that can improve collaboration between health care professionals: health care organization (i.e. the organization of the health care such as leadership and culture), delivery system (re)design (i.e. the organization of care provision for instance by assigning different roles to different people), decision support (i.e. putting evidence based clinical guidelines into practice for example by the introduction of a reminder system), and clinical information systems (i.e. systems to support the information exchange) [11]. Each component encompasses a variety of solutions, the so-called elements, to improve collaboration.

**Table 2:** The solutions proposed to improve the collaboration classified according to the chronic care model*

	Experienced #	Expected #
**Health care organization**	**37**	**9**
Easily approachable informal contact	25	2
Association of professionals	5	NR
Quality management	4	2
Accreditation	1	NR
External stimuli	2	3
Leadership	NR	2
		
**Delivery system (re)design**	**15**	**24**
Changing allocation of tasks	15	24
		
**Decision support**	**23**	**89**
Education		
Education	13	31
Repeating the message	2	5
Establish an image	2	7
Protocols/agreements		
Multidisciplinary	NR	11
Shared protocol	2	12
Agreements	1	6
Checklists	1	3
Meetings		
Multidisciplinary	NR	5
Per discipline	NR	5
Bilateral	2	1
Other	NR	3
		
**Clinical information system**	**10**	**13**
Shared clinical information system	10	13

*Solutions proposed to improve collaboration are categorized under experienced solutions (i.e. the solution is by the interviewee experienced to improve the collaboration in the past) and expected solutions (i.e. the solution is merely based on expectations rather than experiences);

#, number of times reported by AC professionals;

NR, not reported during the interviews by AC professionals.

In addition, we classified the perceived barriers using Cabana's framework. This framework categorizes physicians' barriers to behavioural change in daily care practice [14]. Following Cabana, we categorized the barriers in line with the widely adopted categorization of professionals' knowledge, attitude, and behaviour. It is assumed that improvement of collaboration can be achieved, first by enhancing knowledge, and changing attitudes and behaviour. Since this framework was originally developed to facilitate the implementation of guidelines, we extended the framework to include barriers that did not fit into the categories of Cabana's original framework.

## Results

### Bottlenecks in collaboration

The bottlenecks the interviewees reported concerning the collaboration with other health care professionals were mainly related to the following three themes: lack of knowledge, lack of consensus among professionals, and inadequate information exchange.

First, a lack of knowledge was most frequently mentioned in relation to non-AC specialists and nursing home professionals, and also, but less frequently so, in relation to general practitioners and dentists. Examples included general practitioners who prescribed avoidable interacting co-medication and specialists who failed to take a patient's dosage history into account when prescribing anticoagulants after surgery.

*For example, although a patient was stable with 6 tablets a day, he was discharged with a dosage advice of 4, 2 and 2 tablets for the following three days. Of course, the next INR measurement results will be no good*. [PD 11.2]

The second bottleneck that we found was the lack of consensus as to treatment. Treatment advices differed between specialists as well as between specialists and ACs. As a consequence, patients received conflicting advices from different professionals.

*If a patient, for instance, needs surgery in a hospital outside the region. The protocol used in a hospital outside the region may well differ from ours, since there are numerous different protocols. We don't take any action to find out about the protocols they use, because you won't get the information. Even our hospital has three different protocols*. [PD 2.1]

The third bottleneck in collaboration pertained to the exchange of information. This was experienced as inadequate to various degrees: absence of information exchange (e.g. information about complications or previous dosage is lacking), incomplete information exchange (e.g. forms are not correctly completed), insufficient information because of inadequate forms (e.g. there are shared forms about co-medication but additional important information is lacking on these forms), and loss of information (e.g. dosage advices sent by fax being indecipherable). As a consequence, ACs have to make time-consuming efforts to get the required and correct information.

*Really... well... it is sometimes a real struggle to get the information about a hospital admission. It is and remains hard to get the information... there are a lot of departments and a lot of people*. [PD 2.3]

The three major bottlenecks were interrelated. For instance, some interviewees mentioned that in their experience nursing home professionals had limited knowledge of OAT and hence failed to send relevant information with potentially detrimental effects.

Interviewees indicated that the quality of care is affected by these bottlenecks in three ways. First, patients might use a suboptimal dosage of oral anticoagulants with an increased risk of hemorrhages or thrombosis. Second, the professional's efficiency is negatively affected. To ensure that patients receive optimal OAT, AC professionals need to make time-consuming efforts to get the information they need or to achieve consensus with other professionals. Third, patients become confused, not knowing what to do, because of conflicting advices from different professionals.

### Solutions to improve collaboration

After inquiring about the problems in collaboration between the health care professionals, interviewees were asked how they propose to counter these problems. Overall, the analyses showed that there were no substantial differences between the types of professional (i.e. manager, doctor and nurse) and their proposed solutions for improvement. If any differences were identified between the experienced solutions and the solutions that were merely proposed based on expectations rather than experiences, this is mentioned. Below the proposed solutions are described per CCM component.

#### Health care organization

The following solutions regarding the health care organization were proposed to improve the collaboration: easily approachable informal contacts, involvement of associations of health care professionals, quality management, accreditation, external stimuli, and leadership (table 2). Solutions regarding the health care organization were mentioned far more often by professionals who had experienced that the organization positively affected the collaboration than when it was merely expected to have that effect. Hence, professionals did not incline to reform their own health care organization to improve the collaboration. Considering the health care organization, the solution most frequently proposed was easily approachable informal contact, especially mentioned with regard to both a personal relationship with other professionals and to the hospital setting (e.g. access to the clinical information system, participating in regular meetings, and more frequent contact).

*The cardiologists are basically our major suppliers and we have the advantage that we are located in the same building and participate in the same regular meetings. Consequently, we keep an eye on everything and have the opportunity to discuss what is going on*. [PD 8.3]

#### Delivery system (re)design

All the proposed solutions regarding delivery system (re)design could be roughly divided into two categories. On the one hand, some ACs had or would like to expand their responsibilities by giving dosage advice to hospitals and/or nursing homes.

*Nowadays, we deliver OAT in nursing homes as well as the psychiatric hospital. In the past, the psychiatrists provided the OAT. ... We saw a reduction in the frequency of the INR-checks from once a week to less than that*. [PD 5.2]

On the other hand, some ACs stimulated or would like to stimulate professionals to take on a more active role, for example by giving nurses more responsibilities in measuring INR in nursing homes.

*There is currently one nursing home that measures INR. And I have to say, it is great. We get a lot of more information, like the use of antibiotics... Before, we had to gather the information after we measured a deviating INR*. [PD 3.3]

This development is in line with the aspiration of some ACs to present themselves as expert centres in their region.

#### Decision support

The solutions put forward regarding decision support could be categorized as: education, protocols and agreements, and meetings. Education, especially for non-AC professionals, was mentioned most frequently to improve collaboration. Education included refresher courses, additional medical training within the curriculum for medical doctors, and the exchange of information. Meetings as well as protocols and agreements were, in contrast to education, merely expected rather than experienced to improve the collaboration. Various forms of protocols and agreements were proposed, including multidisciplinary protocols, shared protocols, and agreements and checklists. Several interviewees would like to develop a multidisciplinary protocol in cooperation with all specialists involved. The form of the meetings that were put forward also varied between interviewees: some suggested regular multidisciplinary meetings whereas others proposed occasional bilateral meetings.

*I got enough of structural meetings. I think meetings cost a lot of time and to little effect. Occasional bilateral consultations are much better. It costs me more time, but I think... I always compute the costs when half of those present are too tired to listen*. [PD6.2]

#### Clinical information system

A shared clinical information system was proposed several times as a way to improve collaboration. Whether interviewees proposed this solution on the basis of experience or expectation depended on the AC region as well as on the discipline they referred to. Professionals of ACs located within a hospital mentioned more frequently that having access to the hospital's clinical information system was a good way to improve the collaboration. Yet, some professionals of ACs located outside a hospital had also access to hospital's electronic patient files as AC professionals often fulfil additional tasks for the hospital. With regard to the pharmacist's role in OAT, it was frequently suggested that an alert-system prevent the supply of interacting drugs. The exchange of information between ACs and general practitioners was hardly ever supported by a shared information system.

*Well, we inform the out-of-hours primary care centre about patients with an international normalized ratio (INR) above 8. The INR is a standardized transformation of the prothrombin time to assess the degree of anticoagulation. However, what if a patient has an INR of 7... then the patient has to report about the OAT and that's not always the case.... Yes, in situations like that... it would be a great help if we all had access to a shared electronic patient file*. [PD 5.2]

### Barriers to a successful implementation of proposed solutions

Subsequently, interviewees were asked to explain why the proposed solutions had not as yet been the answer to their problems. The experienced barriers to improve the collaboration are described using the three levels of Cabana's framework: knowledge, attitude, and behaviour [14] (table 3).

**Table 3:** Experienced barriers to improve the collaboration

Barriers	#
**Knowledge**	**9**
**Lack of awareness**	7
**Lack of knowledge**	2
	
**Attitude**	**47**
**Inertia of perious practice/lack of motivation**	23
	
**Lack on outcome expectancy**	
Lack of recognition/AC status	11
Professional autonomy affected	6
	
**Lack of agreement to collaborate**	
Conflict of interests	5
Responsibilities unclear	1
Fear of losing work	1
	
**Behaviour**	**85**
**Lack of time/time pressure**	
Lack of time	14
Time pressure	9
	
**Lack of resources/materials**	
Lack of money	6
Lack of manpower	4
Lack of IT applications	3
Forms hard to fill out	1
	
**Organisational constraints**	
Organisational policy	10
Lack of authority of ACs over non-AC professionals	5
Professionals are not united	7
Turnover of staff in collaborating organizations	12
Many involved professionals	6
Size of organizations	3
	
**Contextual factors**	
Competition between health care organizations	3
Legislation	2

#, number of times reported by AC professionals.

#### Barriers related to knowledge

The two categories proposed by Cabana - lack of knowledge and awareness - were experienced by some of the AC professionals as barriers to the improvement of the collaboration in OAT. First, regarding the lack of knowledge, we classified the mentioned barriers under a lack of available evidence and a lack of knowledge about the available evidence. The lack of evidence about the right treatment protocol was only once mentioned as a limitation to improve the collaboration in OAT (table 3): the interviewee reported to wait for more evidence to gain professionals' confidence. Still, it was noticed that more insight and thereby awareness could be obtained if other professionals gave more insight in the care process, however, this is restricted by limited information exchange. For instance, professionals could not learn from the effectiveness of their suboptimal dosage advice since the treatment and patient outcomes are not exchanged between the hospital and the AC.

Second, the lack of awareness of professionals about the need of collaboration for OAT was a prevalent barrier outside the AC (table 3).

*I think we have a clear understanding of the problems which could be caused; and that problems are caused by poor communication. I think that not everyone is aware of this fact*. [PD 5.3]

Although other barriers were frequently mentioned to hamper the successful implementation of specific proposed solutions, lack of awareness was mostly mentioned to hamper the improvement of collaboration in general. Even though AC professionals were aware of the bottlenecks and consequences because INR measures gave them insight in the quality of care, an increasing awareness of some AC professionals could still be noticed during the interviews.

#### Barriers related to attitude

The following barriers, classified under attitude, were reported: lack of motivation, lack of outcome expectancy (i.e. professionals do not expect positive effects of collaboration), and lack of agreement to collaborate.

First, lack of motivation was the most frequently mentioned barrier (table 3). For example, some interviewees noticed that providing education, which was often proposed as a solution, is not enough to improve the collaboration.

*The training we provided in nursing homes was not very successful. Somehow it failed to stick, people just weren't interested enough*. [PD 17.3]

The same applied to clinical information systems; the care providers need to use it correctly for the system to be successful in improving the exchange of information.

*Specialists have access to the clinical information system of the AC and can see the last dosages prescribed. Nevertheless, it happens that specialists prescribe of caution a much lower dosage than the patient was prescribed before hospitalization. Consequently, the patient has an increased risk for thrombosis*. [PD 8.2]

In addition, analysis of the barriers linked to the proposed solutions showed that the lack of motivation was mentioned as a cause for limited or slow implementation for all solutions (see also additional file 2). Furthermore, a lack of motivation was most frequently mentioned to hamper the successful implementation of protocols and agreements.

Second, the lack of outcome expectancy of collaboration was related to a lack of recognition of OAT and AC status, and other professional's challenge to autonomy. Not having status as an AC was frequently put forward as a barrier to improve the collaboration For instance, an AC director experienced that none of his advices to improve hospital's IT system to improve the collaboration between the AC and hospital were followed. Furthermore, some professionals tended to ignore AC professionals' advice as they experienced this as a threat to their own autonomy and professional expertise. It was suggested, that the expertise of the AC professionals and improved AC status could reduce this experienced threat.

*When we are on the phone and they become aware of our expertise and their own lack of knowledge, the conversation changes. Afterwards we are valued. Some professionals may even contact us for advice in the future. So, in that way, it is probably effective to present oneself an expert, I suppose*. [PD 16.1]

Third, the lack of agreement regarding the way to collaborate was also experienced as an impediment to improve the collaboration in OAT. The barriers identified in this category were conflict of interests (e.g. different preferred balance between hemorrhage and thrombosis), unclear responsibilities, and fear of losing work. These barriers stand in the way of the allocation or reallocation of tasks and the development of agreements and protocols (additional file 2).

#### Barriers related to behaviour

The environmental barriers affecting professionals' behaviour were classified under the following categories: lack of time and time pressure, lack of resources and materials, organisational constraints, and contextual factors.

First, lack of time and time pressure restricted the improvement of collaboration in several ways. Time was a barrier to realize proposed solutions as well as to apply these successfully. AC professionals were motivated to improve the collaboration, yet were restricted by time pressure.

*We should continuously evaluate the chronic care process. How does it go? Could we improve it somewhere? Are we still satisfied with the current agreements? ... Actually we should put more effort in this; however, this isn't daily care practice*. [PD 12.1]

When the proposed solutions were linked with the mentioned barriers, it became evident that especially the exchange of clinical information was restricted by time pressure. In particular, there were agreements to use specific forms to exchange information between health care professionals but time pressure hampered to fulfill the forms.

Second, experienced barriers classified under lack of resources and materials were lack of money, insufficient personnel, and inadequate IT-applications. For instance, reorganisation was mentioned as a reason why activities to improve the collaboration were not initiated.

Third, several organisational characteristics hampered the implementation of proposed solutions: organisational policy, lack of authority of ACs over non-AC professionals, professionals not being united, turnover of staff in collaborating organisations, the number of professionals involved, and the size of the organization (table 3). For example, professionals were hindered to achieve agreements regarding collaboration and task delegation by the lack of representatives of the 300 general practitioners and dentists in certain regions. In addition, AC professionals experienced limited power to influence organisations regarding the great variety of existing protocols. AC professionals reported more than once to follow the dosage advices of other professionals to prevent unnecessary delay.

*We frequently ask the patient whether they received a recommendation and from who, as some doctors and dentists have their own protocols. For example, we don't agree with the policy of a specific hospital... but we could not convince the hospital to change it. They are in charge and they decide. You have to follow their advice; otherwise patients are sent home without an operation*. [PD 12.3]

Fourth, mentioned contextual factors that hampered the improvement of collaboration included the introduction of competition in the market and legislation (table 3). Both hindered to enter agreements. For instance, the competition in the health care market caused a fast shift of home care services which inherently asked frequent modification of the agreements (content and/or change of collaborating organization). Furthermore, competition between ACs hampered the information exchange between some ACs.

*However, nowadays we have competition to contend with, since all three hospitals have their own AC. Consequently, it is also... a matter of survival these days*. [PD 13.2]

## Discussion

This study aimed to gain insight into the needs and mechanism to improve the collaboration in OAT. Therefore, this study identified the bottlenecks in collaboration between health care professionals in OAT, the proposed solutions to overcome these bottlenecks, and the perceived barriers to implement these successfully. Our study revealed that professionals experienced several problems in the collaboration regarding OAT that need to be improved. The most prevalent experienced bottlenecks were lack of knowledge, lack of consensus about OAT among health professionals, and inadequate information exchange between health professionals. The proposed solutions to overcome these bottlenecks were related primarily to the improvement of professionals' decision support, mainly by education. Finally, several attitudinal and behavioural barriers to improve collaboration for OAT were identified, most frequently mentioned were lack of non-AC professionals motivation and lack of time.

As far as we are aware, our qualitative study is the first that explicitly identified the experienced bottlenecks in collaboration in daily care practice for OAT. In line with previous studies on chronic care, our results reveal that collaboration needs to be improved to manage the OAT successfully [15-17]. This study showed that the three identified bottlenecks in collaboration, i.e. lack of knowledge, lack of consensus, and lack of information exchange were experienced to affect the quality of care negatively. The quality of care was seriously affected since these bottlenecks resulted more than once in the use of a suboptimal dosage of oral anticoagulants, inefficient time-consuming gathering of patient information, and patients being confused due to conflicting dosage advices from different professionals.

The proposed solutions given by the interviewees are in line with CCM's suggestions for improvement, such as multidisciplinary meetings and quality management [18]. Especially decision support elements (i.e. education, meetings, and agreements and protocols), were proposed to improve the collaboration. Remarkably, a discrepancy between experiences and expectations revealed regarding the proposed solutions. Formal approaches like protocols and regular meetings were frequently proposed as solution by our interviewees as well as in the CCM literature, while in ACs where the formal approaches were used, these formal approaches were scarcely experienced to improve the collaboration. In addition, solutions regarding the health care organization were frequently proposed, but only if the professional experienced that their organization influenced the collaboration positively. Hence, the potential of the health care organization to improve collaboration seemed to be underestimated by professionals. Professionals are not inclined to reform their own health care organization to improve the collaboration. These discrepancies show the importance to exchange experiences between professionals prior to implementing proposed solutions.

Moreover, AC professionals considered education of other professionals a prerequisite for a successful implementation of other proposed solutions. This is in line with the implementation sequence of other complex interventions [14,19]. As a consequence, although CCM components are supposed to be more effective if they are applied as comprehensive interventions [11,18], we argue that these interventions can be even more effective if the sequence of implementation of these components is taken into account. This is in line with other models of behavioural change that identified knowledge as a prerequisite for acceptance and attitude change [20]. Taking the sequence of implementation into account will result in a reduction of the number of both priorities and changes at the same time which has also been shown to improve the implementation and thereby the effectiveness of the interventions [19,21].

Education for other professionals as well as the implementation of other proposed solutions were also hindered by attitudinal and environmental constraints as is in line with previous studies [14,19,21-23]. Our results revealed that the attitudinal constraints could mainly be related to a lack of motivation in non-AC professionals. Their lack of motivation to improve the collaboration was mentioned as a cause for partial or slow implementation of all proposed solutions. Although the lack of motivation could be due to factors unknown by AC professionals, it is related to a lack of alignment regarding the collaboration between AC professionals and non-AC professionals. Especially alignment regarding chronic care management is needed for successful collaboration [10,13,24]. Alignment of all involved professionals should also encounter the lack of status which can be interpreted as lack of trust. Trust is needed as it is identified as one of the fundaments to collaborate [9,13].

However, reaching alignment is probably also hampered by the lack of status and knowledge. Based on our results it seems likely that easily approachable informal contacts could improve ACs' status and professionals' knowledge as was also suggested by others [9,10]. Furthermore, our results showed that a lack of time and money hampered the improvement of collaboration, since the professionals are focused on specific tasks (e.g. measuring INR, giving dosage advice) instead of the entire chronic care process. This is in line with the financial constraints that were experienced by professionals in improving the collaboration for other chronic diseases [19,25].

Besides general conclusions regarding the needs and barriers to improve the collaboration in OAT in the Netherlands, this study elicited regional differences that should accounted for. First, regions can inspire each other by their differences. For instance, a few ACs were hampered to improve the collaboration because general practitioners were not united. One of the ACs did overcome this barrier by involving the out-of-hours primary care centers in OAT in which all general practitioners of the region are unified. Second, solutions should be adapted to a regional level since complex interventions work best if they are tailored to local contexts rather than completely standardized [26]. Third, our results showed that more discussion and knowledge is needed regarding the variation in treatment protocols between regions, disciplines, and hospitals to improve the quality of care.

The strength of this study is that we systematically identified professionals' experiences with improving collaboration in OAT, since previous research emphasized that the implementation process should be understood to develop an effective and sustainable intervention [4,5,22,27]. However, several limitations of this study should be noticed. First, we only included the perspective of AC professionals because they are the main care providers playing a pivotal role in OAT in the Netherlands. Since regional differences are prevalent we decided to interview AC professionals from different regions instead of interviewing all actors. This could well be the next focus of research as it is of interest to identify the factors that can explain non-AC professionals' lack of motivation that was experienced by AC professionals.

Second, we used Cabana's framework to identify barriers to behavioural change by health care professionals. However, other models could also be utilized and it is up for discussion what model would be best [22]. Since our data were first inductively analyzed and only subsequently categorized using Cabana's framework, the framework did not restrict our analysis compared to other models. Based on our inductive analysis, we added a barrier, lack of status, to Cabana's framework. Third, we used the CCM as theoretical framework since CCM components enable to structure the proposed solutions at practice level, while taking the multidimensional character of collaboration into account. Other collaboration models such as the model of D'Amour could be of additional value in further research when the extent of collaboration needs to be measured and further detailed analysis is preferred. Finally, we only interviewed professionals of 30 percent of the Dutch ACs. Nevertheless, data saturation seemed to be achieved since we did not achieve new insights in the lasts interviews.

## Conclusion

This study revealed that the collaboration in OAT is limited by a lack of knowledge, a lack of consensus among health care professionals on the best possible treatment, and a limited information exchange among health care professionals, and that specific solutions were considered to best improve the collaboration. As education was put forward as a prerequisite for a successful implementation of other proposed solutions the implementation sequence is of importance. Motivational and time constraints need to be addressed to enable collaboration to be improved successfully, which could be facilitated by establishing alignment regarding the collaboration with all professionals involved in OAT.

## List of abbreviations

AC: specialized anticoagulant clinic; CCM: chronic care model; INR: International Normalized Ratio; OAT: oral anticoagulant therapy; PD code: a unique primary document code assigned to every transcribed interview.

## Competing interests

The authors declare that they have no competing interests.

## Authors' contributions

All authors contributed to the design of the study. HWD interviewed all professionals. HWD and ML conducted the data analyses. CAB, BRM WG and GPW supervised the study. All authors contributed to the writing up of this manuscript and approved the final version.

## Pre-publication history

The pre-publication history for this paper can be accessed here:

http://www.biomedcentral.com/1471-2261/11/76/prepub

## Supplementary Material

Additional file 1 **Interview guide**. Document name: Additional file 1*_interviewguide. This document includes the interview guide used by the authors to conduct the interviews for this study*. Click here for file

Additional file 2 **Barriers linked to the proposed solutions to improve the collaboration**. Document name: Additional file 2*_barriers linked to solutions. This document includes a table which outlines the barriers categorized to Cabana's framework linked to the proposed solutions categorized to Wagner's CCM*. Click here for file
