# Supplementary material for: Needs and barriers to improve the collaboration in oral anticoagulant therapy: a qualitative study
Source: BMC Cardiovasc Disord. 2011 Dec 22;11:76. doi: 10.1186/1471-2261-11-76 (PMC3268100; doi:10.1186/1471-2261-11-76)
Supplement: Additional file 1 — Interview guide. Document name: Additional file 1_interviewguide. This document includes the interview guide used by the authors to conduct the interviews for this study. [file 1471-2261-11-76-S1.DOC]

**Appendix 1: Interview guide**

Duration of the interview: about 20 minutes

Interviewer: H.W. Drewes, as Health Scientist trained in qualitative research.

1. **Theme: Cooperation state**

**Question:** How does the cooperation between you and the others take shape at this moment?

**Possible following questions (if not automatically named by the interviewee):**

- With which actors? (dentist, nursing homes, pharmacists (located in and outside the hospital, specialists).
- How is the cooperation organized: protocols, meetings etc. ?
- Intensity of cooperation: contact once a year, months etc?

1. **Theme: Experience with current state of cooperation**

**Question**: How do you experience the cooperation regarding OAT?

**Possible following questions (if not automatically named by the interviewee):**

- Bottlenecks in the cooperation (questioned per actor)?
- Successes in the cooperation (questioned per actor)?

1. **Theme: Preference/ need regarding cooperation**

**Question**: Would you prefer some change regarding the cooperation with other professionals? And if so, in what way?

**Possible following questions (if not automatically named by the interviewee):**

- With which actors? (dentist, nursing homes, pharmacists (located in and outside the hospital, specialists).

1. **Theme: Operationalization of the preference/need**

**Question:** How should this preference regarding cooperation be realized?

**Possible following questions (if not automatically named by the interviewee):**

- Op welke wijze zou u deze samenwerking willen vormgeven?: protocol, overleg, etc.
- Intensiteit: intensiteit van samenwerking

1. **Theme: Explanation of the discrepancy between the preferred and current state of cooperation**

**Question:** Can you explain the discrepancy between your preferred state of cooperation and the current state of cooperation? (*Or if there is no discrepancy: could you explain why other ACs experience a discrepancy between the preferred and state of cooperation?*).
