# Supplementary material for: Needs and barriers to improve the collaboration in oral anticoagulant therapy: a qualitative study
Source: BMC Cardiovasc Disord. 2011 Dec 22;11:76. doi: 10.1186/1471-2261-11-76 (PMC3268100; doi:10.1186/1471-2261-11-76)
Supplement: Additional file 2 — Barriers linked to the proposed solutions to improve the collaboration. Document name: Additional file 2_barriers linked to solutions. This document includes a table which outlines the barriers categorized to Cabana's framework linked to the proposed solutions categorized to Wagner's CCM. [file 1471-2261-11-76-S2.DOC]

**Appendix 2: Barriers linked to the proposed solutions**

| **Proposed initiatives within the CCM** | **Barriers within Cabana's framework** |  | **Illustrative quote** |
| --- | --- | --- | --- |
| **Health care organization** |  |  |  |
| Easy informal approachable contact |  | None mentioned |  |
| Association of professionals |  | None mentioned |  |
| Quality management | Inertia of perious practice/lack of motivation | Inertia of perious practice/lack of motivation | *Well, it depends who is in charge and who happens to be on duty when you come. Some try to help you as well as they can and others won't bother. That is also the case in nursing homes; actually…that is the case for everything.* |
| Accreditation |  | None mentioned |  |
| External stimuli |  | None mentioned |  |
| Leadership | Lack of resources/materials | Lack of manpower | *If you want to collaborate effectively, you need a sufficient number of knowledgeable staff. However, there are too few specialized AC doctors in general.* |
|  |  |  |  |
| **Delivery system (re)design** |  |  |  |
| Changing allocation of tasks | Inertia of perious practice/lack of motivation | Inertia of perious practice/lack of motivation | *Not every department takes part, this depends of the individual specialist.* |
|  | Lack on outcome expectancy | Lack of recognition/AC status | *I would like to be more accepted in our coach/advice role by other specialists.* |
|  |  | Professionals ethics affected | *The specialist has the feeling that something is taken away from him.* |
|  | Lack of agreement to collaborate | Conflict of interests | *Yes, we would like to give dosage advices in the hospital, but other professionals do not prefer that because we do not know what happened with the patient during the day.* |
|  |  | Responsibilities unclear | *The pilots were not performed because of ambiguity regarding the responsibility. …The cardiologist wondered whether we exactly knew patient's status and who is taking the responsibility for the OAT…The clinical status can change frequently in these intra* |
|  |  | Fear of losing work | *Again and again we run into the fear of the clinicians who are afraid that work is taken away from them. Most of them still define the health care process in sub domains and they want to keep control of theirs.* |
|  | Lack of time/time pressure | Time pressure | *The time pressure at the department, I think you will gain time but I cannot convince the other professionals to organize the information exchange in another way.* |
|  |  | Lack of time | *It is also related to what you can handle. We have decided to give priority to the internal quality, more than the external.* |
|  | Lack of resources/materials | Lack of money | *Yes, and if the patient is hospitalized we must not think well that's not our responsibility…but this is also related to our budget..* |
|  |  | Lack of manpower | *We would like to be involved in pilots such as improving the structural information exchange after hospitalizations; however, we have no manpower at this moment.* |
|  |  | Lack of IT application | *In order to give dosage advices to hospitalized patients you have to get all the clinical patient information. However, a shared clinical information system which is prerequisited by our manager is not yet available.* |
|  |  |  |  |
| **Decision support** |  |  |  |
| Education | Inertia of perious practice/lack of motivation | Inertia of perious practice/lack of motivation | *We provide a lunch-session every two year, however, it is less than 10% of the GPs of the region who participate.* |
|  | Lack of time/time pressure | Lack of time | *You can think about education evenings or invite them to come to our AC if they are interested. However, this is very is time-consuming* |
|  |  |  | *We provide lessons at their location, yet they have no time or a lack of motivation.* |
|  | Lack of resources/materials | Lack of manpower | *If you want to collaborate effectively in the network, you need sufficient staff and you have to have the knowledge as leader. However, there are too few specialized AC doctors in general.* |
|  | Organisational constraints | Professionals are not united | *Professionals cannot be reached at once to inform them or to educate them. For instance in a nursing home, you have 300 patients and 300 GPs so to speak.* |
|  |  | Turnover of staff in collaborating organizations | I think we always have been overtaken by events because when a new assistant physician has just been educated, he/she leaves and we have to train the next that starts working at that department. |
|  | Contextual factors | Competition between health care organizations | *The exchange of information is somewhat limited by the introduced competition between ACs. We try to survive as AC within a region where three ACs operate.* |
| Meetings | Lack on outcome expectancy | Professionals ethics affected | *And dentists are worse coordinated than GP's, you will have to speak to each one of them. And each of them will make their own decisions. I do not experience problems in personal contacts.* |
|  | Lack of time/time pressure | Lack of time | *The current GP wants to stop participating in our AC board, but it is hard to replace him. GPs in general have less time to do these kinds of things.* |
|  | Organisational constraints | Professionals are not united | *Its not so easy in daily care practice. There are many professionals which need to be invited (100 GPs, 100 dentists, 20 physicians of the nursing home etc); some are not united (e.g. dentists) and others are united in different groups (still 5 GP groups)* |
| Protocols/ agreements | Knowledge/ lack of awareness | Knowledge/ lack of awareness | *Well, I still had some questions after certain study results were published. You want to present all the information at onxe to the other professionals and that's hard for me. I have the feeling that we have to have considered everything before we can dis* |
|  | Inertia of perious practice/lack of motivation | Inertia of perious practice/lack of motivation | *We developed a protocol regarding surgery. That is electronic available for every professional. Nevertheless, the most idiot dosage advices are still given because they don't check the protocol. The protocol is good and is known ….I can't trace why.* |
|  | Lack on outcome expectancy | Lack of recognition/AC status | *Every time we are hindered, well very hindered, by the fact that not every specialist is convinced that the AC has the knowledge.* |
|  |  | Professional ethics affected | *Even the specialists cannot reach consensus sometimes, not to mention that a outsider is allowed to decide the dosage advice.* |
|  | Lack of agreement to collaborate | Conflict of interests | *We always talk about conflict of interests. One professional prefers another dosage advice than another one. You cannot create one protocol which includes these conflicting preferred dosage advices.* |
|  | Lack of time/time pressure | Lack of time | *However, if you just send these protocols it will not work. You have to have a good quality system that needs to be updated.* |
|  |  | Time pressure | We have agreements...however...if a GP works under high time pressure, I can imagine that he will not directly complete and send the form to the AC. |
|  | Lack of resources/materials | Lack of money | *We have thought about it to do this with the Medical-Board, but you have to question yourself how much money and time you want to spend on it.* |
|  |  | Forms hard to fill out | *It is hard for them to fill out the agreed forms.* |
|  | Organisational constraints | Lack of AC's power regarding non-AC professionals | *Yes, our specialized nurses really want the best for their patients. However, they cannot force specialists to follow the protocol.* |
|  |  | Professionals are not united | *It would be easier to make agreements if there was a care chain platform. However, at this moment we frequently contact the individual professionals to realize/ update agreements.* |
|  |  | AC is too small | *It is hard for the AC to arrange a meeting with all involved actors, especially as small AC.* |
|  |  | Turnover of staff in collaborating organizations | *Also in the nursing homes, there is a continuous change in personnel.* |
|  |  | Many different involved professionals | *In our region, it is difficult because we have several hospitals, which makes it more complicated to realize agreements.* |
|  |  | Organisational policy | *And if we refer to the agreements made with the nursing home after a nurse did not live up to our agreement, they answer that its not their choice but it is the policy of the nursing home.* |
|  |  | Organisation size | *Especially in larger organisations where one doesn't know what the other has done and what should be done…* |
|  | Contextual factors | Legislation | *The legislation is frequently changed, for instance regarding the home care services which were not allowed to fill the pill box anymore. As a consequence, it is hard to reach consensus.* |
|  |  | Competition between health care organizations | *We have a lot of agreements with the nursing home services. However, its results are limited because it differs yearly with which company you have to cooperate with. This started when the competition between home care service was introduced. Yes, it is hard for AC's to enter into agreements these days.* |
|  |  |  |  |
| **Clinical information system** |  |  |  |
| Shared clinical information system | Inertia of perious practice/lack of motivation | Inertia of perious practice/lack of motivation | *It is a matter of two ticks in the computer, one on and one off. It's their decision to switch this option off.* |
|  | Lack of outcome expectancy | Lack of status | *I try to solve crucial problems as director; I participate in structural meetings for two years but in the end the hospital developed an information system for themselves and our question was ignored.* |
|  |  | Lack of time | Structural information exchange with specialists implies a lot of work, much correspondence and also unuseful correspondence. Maybe that's the problem…that we receive a lot of unnecessary information. |
|  |  | Time pressure | We have agreements...however...if a GP works under high time pressure, I can imagine that he will not directly complete and send the form to the AC. |
|  | Lack of resources/materials | Lack of money | *Of course, also money is needed to realize these interventions. You can just organize clinical lessons but clinical information systems...* |
|  | Organisational constraints | Lack of AC's power regarding non-AC professionals | A shared computerized clinical information system would be perfect, however, someone else has to realize this and I cannot force this person. |
